# MineralMate: A standalone MATLAB-based aide for the magnetic separation of minerals

**DOI:** 10.1016/j.heliyon.2022.e10411

**Published:** 2022-08-27

**Authors:** Bowman Samuel, Hnatyshin Danny

**Affiliations:** a Department of Earth and Atmospheric Sciences, University of Alberta, 1-26 Earth Sciences Building, Edmonton, AB, T6G 2E3, Canada; b Marine Chemistry and Geochemistry Department, Woods Hole Oceanographic Institution, 266 Woods Hole Rd., MS #25, Woods Hole, MA, 02543, USA; c Department of Geology and Geography, West Virginia University, 330 Brooks Hall, 98 Beechurst Ave., Morgantown, WV, 26506, USA; d Irish Centre for Research in Applied Geosciences, University College Dublin, Belfield, Dublin 4, Ireland

**Keywords:** Frantz, Geochemistry, Geochronology, MATLAB, Magnetic separation, Mineral separation

## Abstract

MineralMate is a standalone MATLAB-based program designed to optimize the workflow associated with the magnetic separation of minerals. For nearly every bulk geochemical analysis some amount of mineral separation must occur, and the use of an electromagnetic separator is ubiquitous and considered as standard practice in many fields. Despite the commonality in which magnetic separation is used, there are considerable shortcomings. Electromagnet overheating and composite mineral grains are frequently encountered, as well as poorly constrained mineral behavior. These complications ultimately reduce the quality of downstream geochemical data. MineralMate is designed to alleviate these shortcomings by quickly and efficiently producing a magnetic separation workflow allowing the user to: (1) identify and compare optimal recovery ranges for different minerals from a bulk mineral assemblage, (2) identify the parameters on a conventional magnetic separator required to magnetically separate composite grains, (3) create/update user-specific magnetic susceptibility databases through empirical data collection, and (4) utilize an alternative magnetic separation equation.

## Graphical abstract

**Figure:**
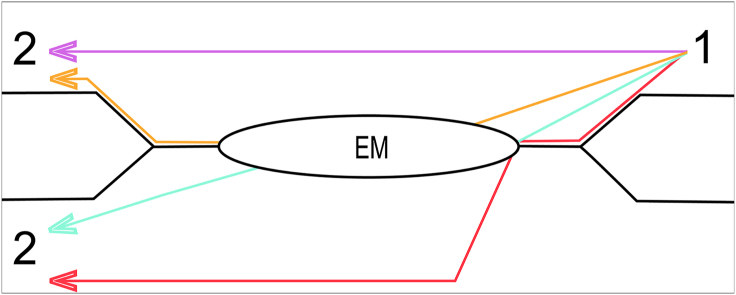

## Teaser

Frantz; Geochemistry; Geochronology; MATLAB; Gagnetic separation; Mineral separation.

## 1 Introduction

The physical separation of minerals from their host rocks and the subsequent creation of purified mineral concentrates is vital for many types of geologic and geochemical analyses. A standardized approach to mineral separation is therefore beneficial for studies that require the efficient extraction of trace mineral phases (e.g. U–Pb geochronology; Weislogel et al., 2016) or use bulk geochemical methods that are sensitive to mineral impurities (e.g. sulfide Re–Os geochronology; Hnatyshin et al., 2020). Physical separation of economically important materials (e.g. ores), also utilizes the same (Chelgani et al., 2015) or similar (Jordens et al., 2013) methods used in scientific research.

In these cases, extracting the desired mineral requires strategically taking advantage of a mineral’s unique physical and chemical properties through specialized mineral purification techniques. Common examples include magnetic separation, heavy liquid separation, or gravitational hydrodynamic separation using a Gemini table or Knelson concentrator (e.g. Majumder et al., 2007)). Industrial or large-scale operations may also incorporate additional separation techniques such as froth flotation (Abaka-Wood et al., 2019b), or leaching techniques, such as bioleaching of Cu and Zn (Williamson et al., 2021), or leaching of Au by S (Sun et al., 2020). Abaka-Wood et al. (2019a; 2019b) note the importance of magnetic separation as a pre-concentrating step prior to downstream froth flotation. While geochemical analyses require pure mineral separates for high-quality datasets, there is a paucity of mineral separation protocols for this work. This observation has also been made by Blackburn et al. (2007), noting that geochronological research (in particular) neglects the importance of mineral separation. Without a well-defined and community-accepted protocol, there is a risk that the chosen methodological approach may produce a lower yield of desired mineral grains, and/or higher proportions of impurities in collected mineral separates. MineralMate is designed to address the magnetic separation step within the larger mineral separation framework. MineralMate seeks to quickly and efficiently produce a magnetic separation workflow by allowing the user to: (1) identify and compare optimal recovery ranges for different minerals from a bulk mineral assemblage, (2) identify the parameters on a conventional magnetic separator required to magnetically separate composite grains, (3) create/update user-specific magnetic susceptibility databases through empirical data collection, and (4) utilize an alternative magnetic separation equation.

## 2 Materials and methods

### 2.1 Fundamental Principles of Magnetic Separation

When a bulk assemblage composed of different mineral particles is exposed to an external magnetic field, the behavioural differences between these minerals can be exploited. The behaviour that a particle exhibits is dependent upon its specific [mass] magnetic susceptibility (K_m_). This parameter is specific to each mineral species, and is the quantifiable degree to which said mineral is magnetized by an external magnetic field. Magnetic susceptibilities in practice are typically positive (e.g. paramagnetic and ferromagnetic minerals), but can be negative in impurity-free diamagnetic minerals (e.g. quartz, calcite, pyrite). Within individual particles, minor elements, trace elements, and crystal structure can cause variations in K_m_ (Burgardt and Seehra, 1977; Dekkers, 1988; Rosenblum and Brownfield, 2000; Chelgani et al., 2015). If a particle is mineralogically heterogeneous (contains impurities)-particularly magnetite or pyrrhotite-the exhibited behaviour when exposed to an external magnetic field can be dramatically affected.

**Figure 1:**
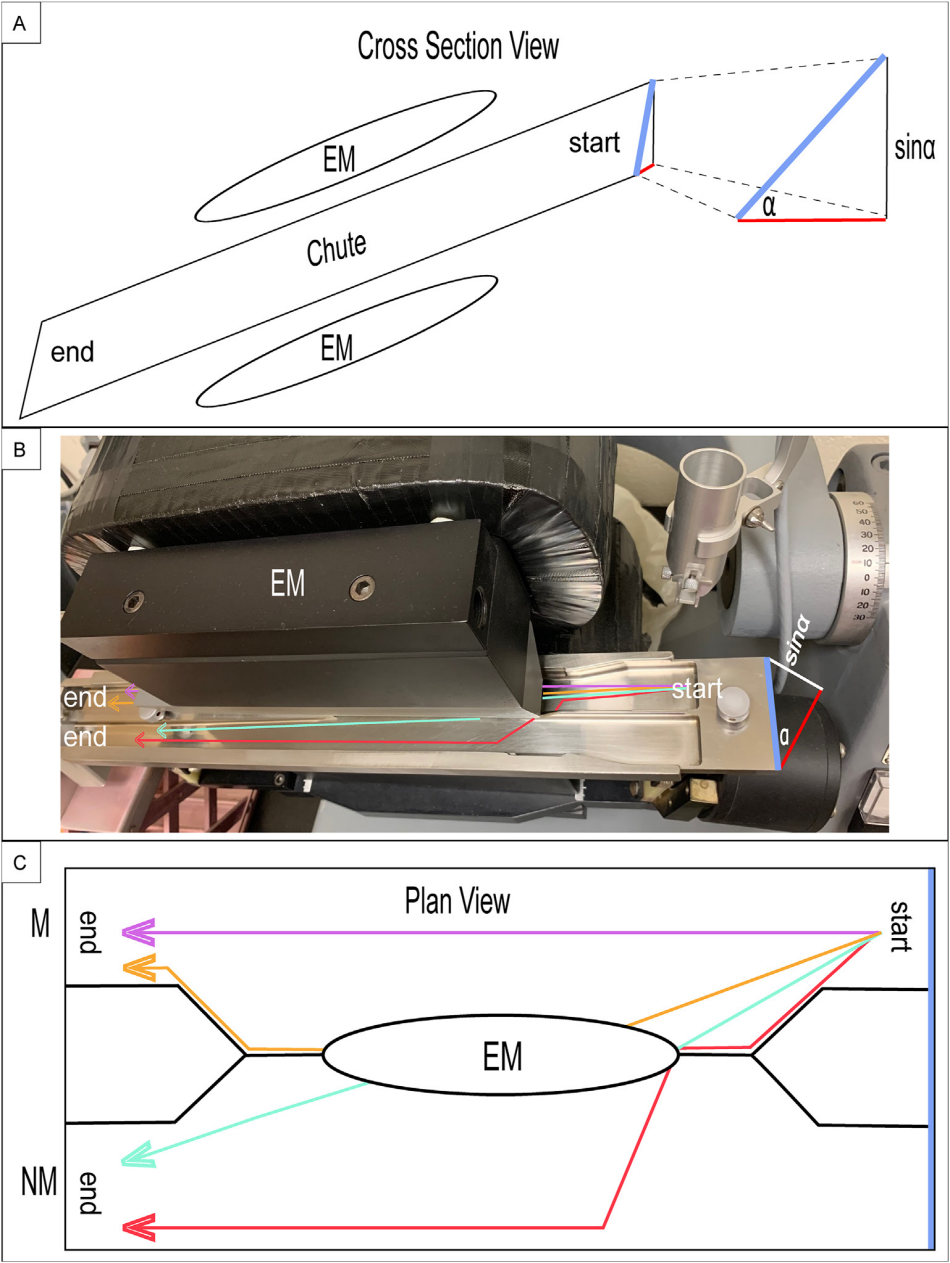
The hypotenuse of sinα is indicated by the thick blue line found on each subpane. The thick red line on the right triangle (cosα) is parallel to the benchtop. Therefore, physically, the thick blue line represents the magnetic separator chute, while the thick red line represents the benchtop. A) Simplified cartoon of an electromagnetic separator. An assemblage of particles is introduced at the top of the chute (start). The particles then travel the full length of the chute, passing through the magnetic field exerted by the electromagnet (EM) and are collected (end). Note that the side slope (α) is angled towards the operator under normal use. B) Photograph of Frantz LB-1, with hypothetical particle paths. C) Cartoon plan view showing magnetic and non-magnetic particle paths. The terminus of the chute (end) may either comprise magnetic (M) or non-magnetic (NM) particles.

Geology and geochemistry labs employ magnetic separators (a Frantz LB-1 was used in this study and described herein) to concentrate a desired mineral from a bulk mineral assemblage. The magnetic separator consists of a vibrating inclined chute that is placed between two poles of an electromagnet that directs the sample into one of two collection cups (Figure 1). The electromagnet, applies an electromagnetic force in competition against the gravitational force. In the typical case where K_m_ > 0 the “non-magnetic” collection cup collects any material where the force of gravity overcomes the attractive force applied by the magnet. The “magnetic” collection cup collects the remaining material. The ratio between the magnetic force, defined by the current (I) powering the electromagnet, and the gravitational force defined by the side slope of the chute (α), allows mineral separation over a large range of K_m_. The equation that has been provided by numerous authors that shows this relationship is of the form:

(1) $$K_{m}=\;\frac{\beta sin\left(\alpha \right)}{I^{2}}\;10^{-6},$$

where the constant β has been estimated to be 19.2 by McAndrew (1957) and 20.6 ± 0.7 from Nesset and Finch (1980) and a value of 20.8 ± 0.9 from this study (See Appendix 1.1 for further discussion). However, Eq. (1) has been suggested by previous studies to only be accurate at currents ≤ ∼1 A. Flinter (1959), for example states that currents over 1.3 A should be avoided. A more robust relationship is provided by Eq. (2), shown below and derived in Appendix 1.2, is based on the empirical dataset from McAndrew (1957) which was calibrated up to ∼1.6 A, and may remain robust up to the maximum current produced on a Frantz magnetic separator (∼2.0 A).

(2) $$K_{m}=sin\;\left(\alpha \right)\;*10^{-6}{\left(\frac{I}{4.7884}\right)}^{\frac{-1}{0.5142}}$$

K_m_ obtained from Eq. (2) are similar to those obtained using the familiar from in Eq. (1), but can be applied with more confidence at higher currents. For example, the K_m_ of forsterite from Eq. (1) (using β of 20) range from 3.24 × 10^−5^ to 3.59 × 10^−6^. The K_m_ from Eq. (2) range from 3.23 × 10^−5^ to 3.82 × 10^−6^. If the K_m_ from Eq. (1) are recalculated using β of 20.8, the values range from 3.36 × 10^−5^ to 3.74 × 10^−6^. The proximity of these K_m_ to the conventional K_m_ values obtained using β of 20 and/or Eq. (1) indicate that both Eq. (2) and β of 20.8 are reasonable. Additional discussion and analysis of all minerals in the Database is provided in Appendix 1.3.

Particle grains that are introduced to the magnetic separator typically have an average diameter of ∼125 μm (Rosenblum and Brownfield 2000). The particle grains used in the example below (Section 3.1.3) are of this approximate size. Magnetic susceptibility in ferromagnetic and antiferromagnetic minerals, may exhibit shape (Carmichael, 1982) and size-dependent hysteresis (Carmichael, 1982; Hunt et al., 1995). Temperature and pressure (Carmichael, 1982; Hunt et al., 1995) may also affect K_m_, however since mineral separation is routinely performed in a controlled laboratory environment at room temperature, then, as Hunt et al. (1995) note, these effects are negligible. Powdered samples are not typically introduced to the magnetic separator due to the inability to orient to the electromagnetic field (Nesset and Finch, 1980). Since these pressure and temperature effects are minimal, MineralMate was designed for magnetic separation occurring at STP and for non-clumping grains (non-powders), which freely move along the chute (Figure 1).

## 3 Results

### 3.1 Features and applicability of MineralMate

**Figure 2:**
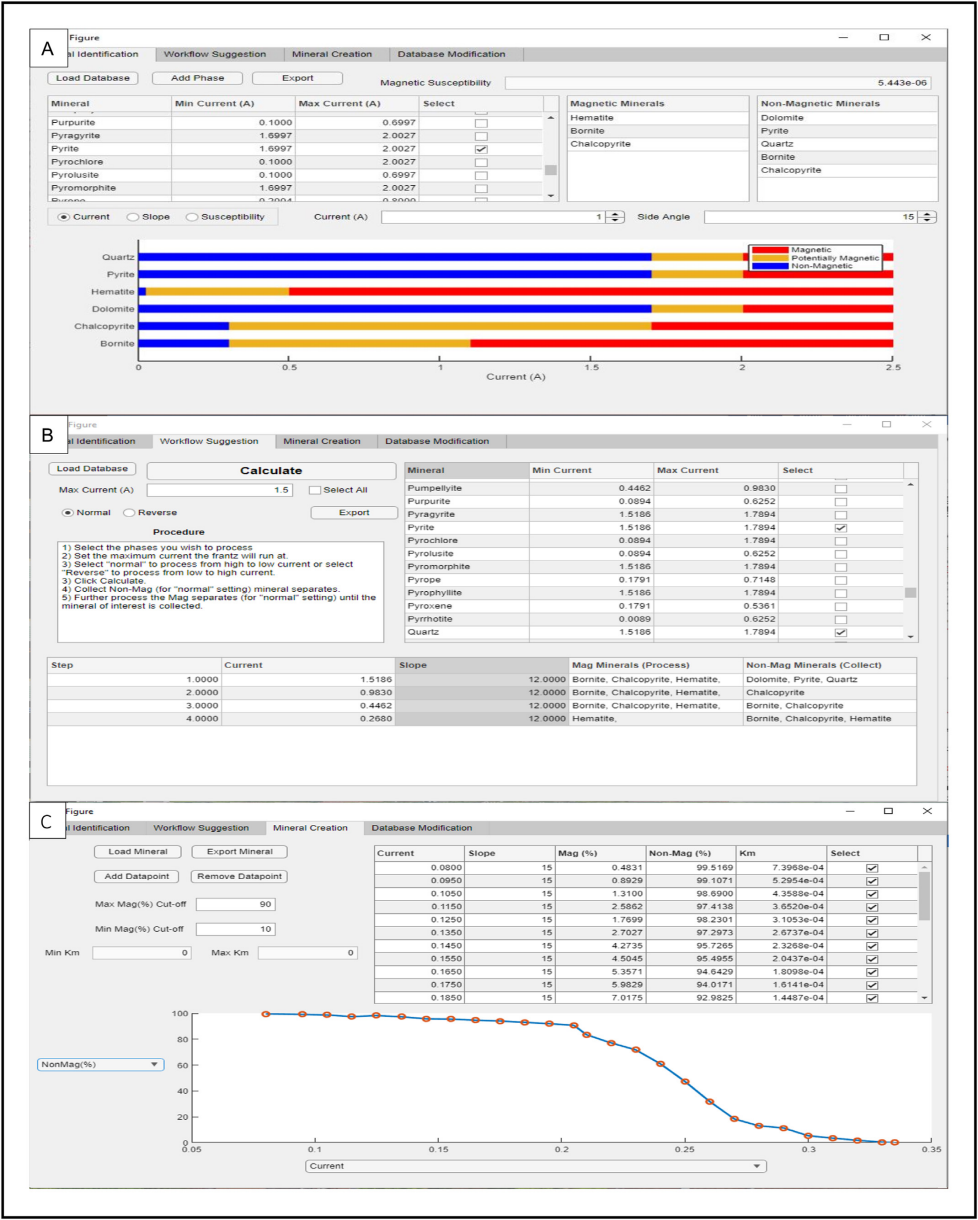
A) Program screenshot of tab 1, where the user can visualize the magnetic behavior of different particles at specific operating conditions. B) Program screenshot of tab 2, where MineralMate creates the optimized workflow for the selected minerals. C) Program screenshot of tab 3, where the user may calculate the K_m_ ranges of a mineral by measuring the recovery at different operating conditions.

The primary application of MineralMate (Figure 2) will be to (1) solve the forward problem whereby the user wishes to predict the recovery range of a specific mineral (Figure 2B); or (2) solve the inverse problem, whereby the user has separated particles at a specific K_m_ and seeks to constrain and identify the collected particles. MineralMate addresses these goals by allowing the user to create workflows for magnetic separation based on Eq. (2) and the K_m_ databases of Rosenblum (1958) and Rosenblum and Brownfield (2000), as well as personalized databases that are created within MineralMate (Figure 2C). MineralMate was designed in MATLAB, and to increase accessibility, MineralMate has also been compiled into an Executable Version and is included as Supplementary Material Data 1. The Source Code is included in Supplementary Material Data 2. Finally, a Database, User Manual, and Example file (Section 3.1.3) are also available as Table 1, Data 3, and Table 2 respectively, in the Supplementary Material. These same files are also available at https://github.com/MineralMate-Program.

#### 3.1.1 Database translations

Previous workers Rosenblum (1958) and Rosenblum and Brownfield (2000), have determined the K_m_ values for over 350 minerals by varying I at α = 15°. However, by fixing α the tabulated values are limited in their flexibility. Given that Frantz electromagnets overheat at high currents (>1.7 A) it is beneficial to determine how to manipulate α to maintain a lower I at a particular K_m_. MineralMate allows for users to rapidly identify the equivalent combinations of α and I by fixing K_m_ and solving for either α or I in Eq. (2). The default K_m_ values in the Database are calculated using the currents reported in the full range for each mineral of Rosenblum (1958) and Rosenblum and Brownfield (2000). Therefore, MineralMate determines the recovery range in accordance to the accepted data from these workers. The user may optimize the recovery range to reflect empirical data (See Section 3.1.3).

#### 3.1.2 Mineral separation workflows

**Figure 3:**
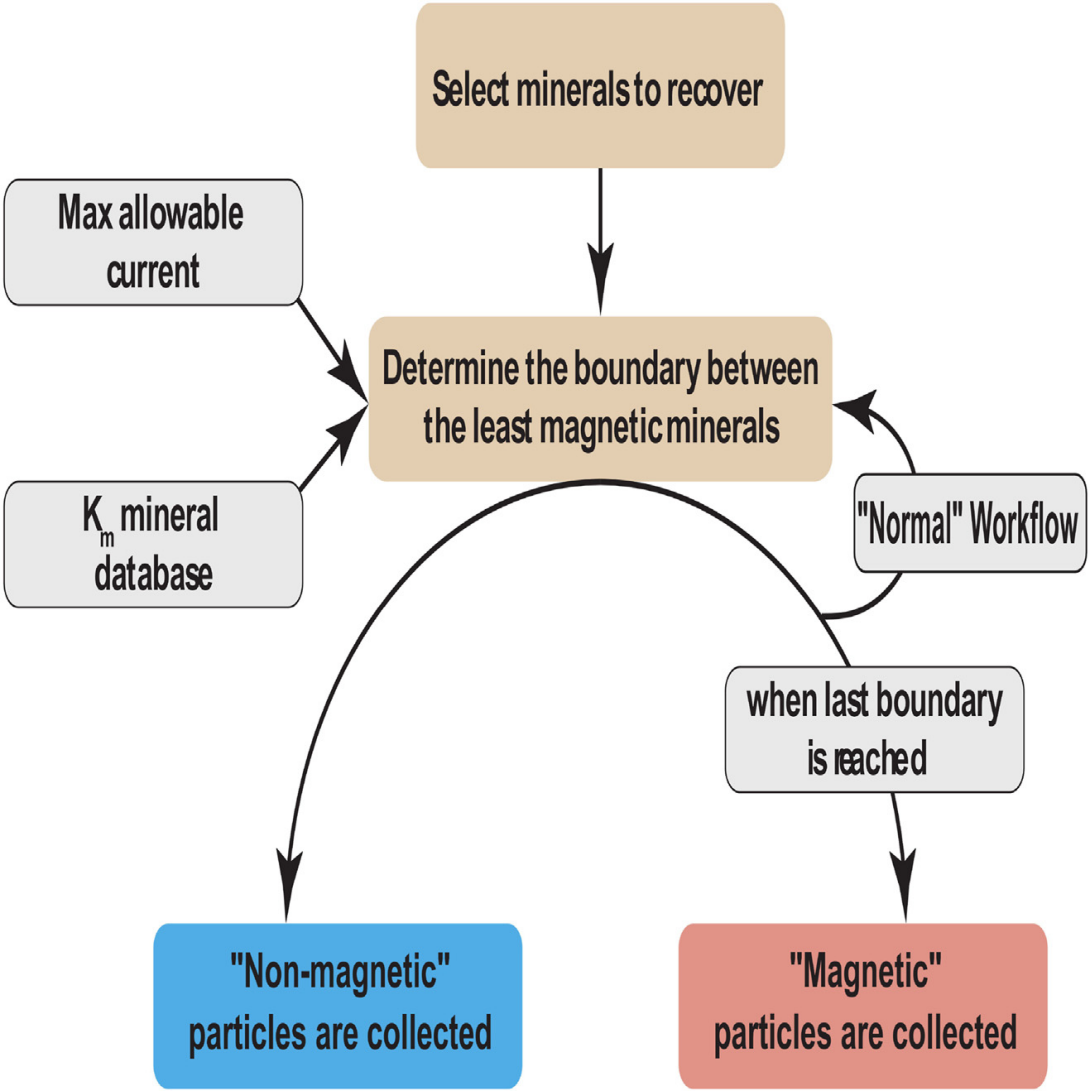
Illustration of the algorithm used in MineralMate to create the workflow (“normal workflow”) in Figure 2B.

One of the primary functions of MineralMate is to use the known K_m_ values of minerals within a bulk sample and provide a workflow that will best isolate the constituent minerals from the bulk assemblage (Figure 3). This is illustrated in the following hypothetical example in Figure 2 with six minerals (bornite, chalcopyrite, pyrite, hematite, dolomite and quartz) that can be partially extracted from each other using magnetic separation based on their differences in K_m_. MineralMate provides a suggested workflow (see attached User Manual) by first comparing (any) overlapping K_m_ ranges of different minerals (Figure 2A) and then selecting the ideal order of extraction by choosing the appropriate α and I values (Figure 2B) that minimizes overlap. The user can set important practical parameters, including the order of extraction (i.e. are high or low K_m_ minerals extracted first) and the maximum current allowable on the Frantz to minimize overheating effects. In this example there are 4 recovery steps required and if followed would produce five separates (Figure 2B): (1) hematite, (2) bornite, (3) chalcopyrite + bornite, (4) chalcopyrite, and (5) dolomite + quartz + pyrite. Due to overlapping ranges in K_m_ values in this example it would be impossible to magnetically separate dolomite, quartz, and pyrite, assuming the K_m_ values used are accurate.

#### 3.1.3 User created databases

The databases of Rosenblum (1958) and Rosenblum and Brownfield (2000) comprise 379 minerals, of which their respective K_m_ has been determined and compiled. The observed K_m_ for a specific mineral from a bulk whole rock is expected to fall within the total range provided by these workers. To supplement existing databases, MineralMate is designed to calculate, save, and export K_m_ values calculated from user entered I and α used during mineral separation. For example, empirical results of a nearly monomineralic forsterite sample are provided below. The experiment is explained for other users wishing to determine the K_m_ value of either a new mineral, or to update a mineral already in the Database (see Table S1).

1. Maintain a constant α throughout the separation process
2. Begin at I = 0 A, introduce sample to the top of the chute
3. Measure the magnetic mass % and the non-magnetic mass % of the total sample (non-magnetic mass % = 100) using a balance
4. Recombine the magnetic and non-magnetic concentrates, mix well
5. Increase I to 0.1 A, introduce sample to the top of the chute
6. Repeat Steps 3–5 until all particles enter the magnetic section of the chute (magnetic mass % = 100)

Enter the experimental parameters into a data table and upload to MineralMate (see Table S2). In this example, the curve in Figure 2C is a CDF plot of current on the x-axis, and non-magnetic mass % on the y-axis. The CDF plot displays large tails towards both extremes of current-resulting in an S-shaped CDF plot. It is possible that the cause of these tails is indicative of multiphase particles (the high current tail likely contains a mineral phase with K_m_ < K_forsterite_, while the low current tail likely contains a mineral phase with K_m_ > K_forsterite_). To avoid interference of these tails in evaluating the K_m_, MineralMate allows the user to select the domain that best constrains a linear relationship-excluding both extremes. In Figure 2C, data points are excluded if the magnetic mass % is less than 10 %, or is greater than 90 %. Therefore, 10 % < Non-magnetic mass % < 90 %, and is used to evaluate the sample K_m_. It is assumed that at these extremes of the K_m_ distribution, the purity of the mineral sample likely worsens (the tail K_m_ is attributed to composite grains). The cutoff is flexible and can be made in accordance to observations made regarding sample purity from an optical microscope. This decrease in sample purity (mixed with another mineral phase) will manifest as K_m_ much greater or much lower than that of the median K_m_ (where it is assumed that the sample is the most monomineralic and thus, the best representation of the "true" K_m_). If, however, as in this example, there are no datapoints existing at exactly 10% and 90%, then MineralMate linearly interpolates between the two nearest bounding points (i.e. 90.7% and 83.5% for the 90% cut-off) to calculate the K_m_ values. The K_m_ values in this example range from 5.96 × 10^−5^ and 1.18 × 10^−4^. These values can then be added to the Database for future use.

By explicitly defining how the “best recovery range” is determined, MineralMate improves upon existing databases, which provide only a qualitative “best recovery range”, defined as “contain[ing] the greatest amount” (Rosenblum and Brownfield, 2000). We encourage users to upload their empirically determined K_m_ Database to the open access spreadsheet at https://github.com/MineralMate-Program. This link contains the converted K_m_ information from Rosenblum and Brownfield (2000) and Rosenblum (1958), as well as the forsterite data in this example.

#### 3.1.4 Composite particles

**Figure 4:**
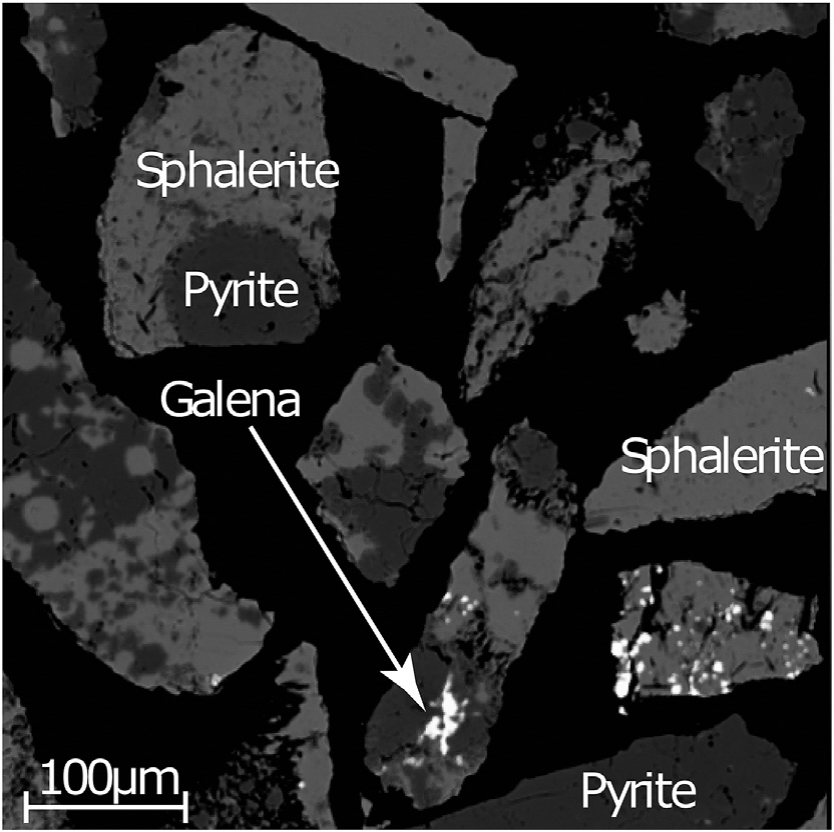
Example of a mineral separate that may be processed through a magnetic separator. In the field of view both monomineralic (sphalerite) and composite particles (e.g. sphalerite + pyrite) are present in the sample.

Physical disaggregation of geologic materials commonly results in fractured particles that do not align with precise mineral-specific grain boundaries or cleavage planes. This physical disaggregation therefore results in mineralogically heterogeneous particles (Figure 4). The K_m_ of these particles will be a linear combination of its constituents. For a multiphase particle, the magnetic susceptibility will be a weighted average, denoted as K_Composite_ and expressed as

(3) $$K_{Composite}=aK_{A}+bK_{B}+cK_{c}+,\ldots ,nK_{N}$$

where a, b, c,…,n are the mass fractions of the phases present (n = 1, or 100 %), while K_A_, K_B_, K_C_,…,K_N_ are the K_m_ values for the respective phases in the composite particle. The constituent components of a composite grain may be quickly verified using an optical microscope. If the composition of the mineral phases present are representative of the composite grains in the sample, then this information may be entered into MineralMate. For example, a composite grain consisting of four unique mineral phases in equal fractional abundance, then n = 0.25 (or 25 %) for each K_N_. MineralMate offers users the ability to create and add multiphase particles to their Database by solving for K_Composite_ in Eq. (3) (see Figure 8 in the User Manual). If the composite grain contains or is likely to contain a desirable mineral phase, the composite grain should be retained and collected alongside the desirable mineral separate. The number of partial desired grains could be successively increased by repeating the separation process. As the mineral heterogeneity (N phases) increases, the n of the desired phase decreases, and K_composite_ is adjusted accordingly. The composite grains may then be reprocessed through the magnetic separator at the new side slope and current suitable for extraction. This approach increases the total number of whole or partial desired grains. The concept outlined above could be scaled-up for industrial purposes, as Abaka-Wood et al. (2019b) state that the yields obtained from froth separation extractions are reduced because composite grains containing a desired phase are not collected. Downstream analyses where this may be an appropriate approach include spatial spot analysis such as LA-ICP-MS or EPMA. These analyses allow for specific regions of interest to be selected, and the undesirable regions on a composite grain may be avoided in-situ. Alternatively, if the downstream analysis involves dissolution such as ID-TIMS, then it is advantageous to avoid composite grains entirely as they would contaminate the isotopic signature of the analyte. In this scenario, the composite grains should be screened-out and not included with the desired grains.

## 4 Discussion

### 4.1 Improved efficiency of magnetic separation

MineralMate was designed to have an intuitive and easy to use interface that can increase the efficiency of magnetic separation through idealized workflows and optimized working conditions. The operating conditions suggested by MineralMate will reduce the probability of the electromagnet overheating by offering equivalen**t** operating conditions at lower current. This allows continuous operation by minimizing cooldown times. MineralMate also provides predictability regarding mineral output, therefore limiting redundant steps during mineral separation.

### 4.2 Example application

One of the primary applications of MineralMate will be providing workflows for geoscientists preparing samples for bulk geochemical methods. For example, sulfide Re–Os geochronology (e.g. Hnatyshin et al., 2020), as well as Lu–Hf and Sm–Nd geochronology (Scherer et al., 2000) are bulk geochemical methods that often require >100 mg of a specific mineral species (e.g. pyrite, garnet). Additionally, non-bulk samples consisting of single particles are routinely used in U–Pb zircon geochronology (e.g. Weislogel et al., 2016).

Creation of multiphase particles during the crushing of material may be extensive (Figure 3) and MineralMate allows the user to identify what conditions will extract composite particles. Reducing the number of composite particles collected during sample preparation is especially important for geochronologic studies because impurities may lead to apparent ages that do not reflect the true age of mineralization (Bowman, 2019; Hnatyshin et al., 2020).

MineralMate streamlines magnetic separation procedures by providing a standardized protocol for the calculation of K_m_ values-improving upon the qualitative evaluation used in some publications (e.g. Hnatyshin et al., 2020; Paradis et al., 2020). Documenting K_m_ values is recommended in any study that uses magnetic separation because this documentation allows for independent analysis, scrutiny, and standardization of the magnetic separation procedure.

## 5 Conclusions

The MATLAB-based, but standalone program MineralMate, provides a flexible, and easy to use platform that is designed to enable researchers to maximize their mineral separation efficiency when using a conventional, laboratory-type magnetic separator. MineralMate allows the user to create a high precision workflow that will maximally recover minerals based on existing K_m_ databases of over 350 minerals, as well as providing a method to create personal Databases. The operating conditions suggested by MineralMate are optimized to provide the lowest currents in order to minimize the risk of overheating the electromagnet. MineralMate provides an intuitive platform to visualize and calculate the expected behavior of different mineral phases. Therefore, inverse problems can also be considered, where the mineral species of collected separates can be investigated based on the recorded operating conditions. MineralMate along with user-created Databases can be downloaded and shared to the global community for iterative improvement and expansion.

## Declarations

### Author contribution statement

Bowman, Samuel; Conceived and designed the experiments; Performed the experiments; Analyzed and interpreted the data; Contributed reagents, materials, analysis tools or data; Wrote the paper. Hnatyshin, Danny: Conceived and designed the experiments; Performed the experiments; Analyzed and interpreted the data; Contributed reagents, materials, analysis tools or data; Wrote the paper.

### Funding statement

This research did not receive any specific grant from funding agencies in the public, commercial, or not-for-profit sectors.

### Data availability statement

Database data is available with submission as Supplementary Data. Also, we plan to add a User Database on the MineralMate Github account.

### Declaration of interest’s statement

The authors declare no conflict of interest.

### Additional information

Supplementary content related to this article has been published online at https://doi.org/10.1016/j.heliyon.2022.e10411.

## Appendix A Supplementary data

Table S2 Table S2

MineralMate User Manual.pdf MineralMate User Manual.pdf

MineralMate.exe MineralMate.exe

readme.txt readme.txt

MineralMate Appendix.docx MineralMate Appendix.docx
