# Supplementary material for: MineralMate: A standalone MATLAB-based aide for the magnetic separation of minerals
Source: Heliyon. 2022 Aug 27;8(9):e10411. doi: 10.1016/j.heliyon.2022.e10411 (PMC9459427; doi:10.1016/j.heliyon.2022.e10411)
Supplement: MineralMate User Manual.pdf [file mmc3.pdf]

MineralMate User Manual – V1.0

A user guide for; MineralMate: A standalone MATLAB-based aide for the magnetic separation of minerals

Authors: Samuel Bowman and Danny Hnatyshin

Any use of this software must reference:

Bowman, S. and Hnatyshin, D., 2022, MineralMate: A standalone MATLAB-based aide for the magnetic separation of minerals, Heliyon, vX, eXXXXX

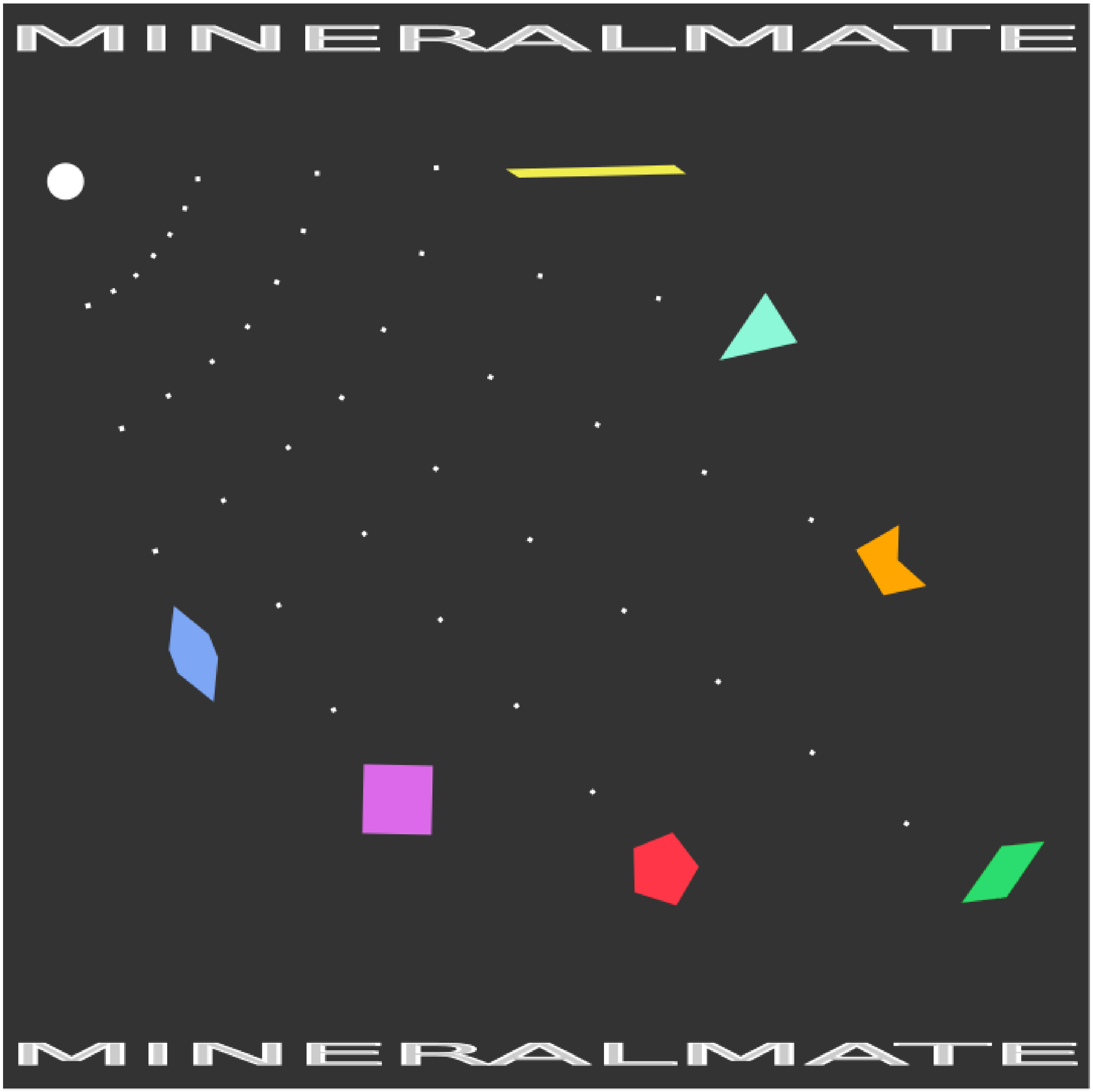

Table of Contents

Installation**………………………………………………………………………..1**

Operation**………………………………………………………………………….2**

Mineral Identification……………………………………………………………3

Workflow Suggestion…………………………………………………………….5

Mineral Creation…………………………………………………………………6

Database Modification…………………………………………………………...9

*Add/Delete Phase***……………………………………………………………….9**

*AddComposite Phase***………………………………………………………….10**

Installation

Download and install MATLAB 2020a (other versions have not been tested) and run the .mlapp code.

OR

Download and run the MineralMate.exe file from one of the available repositories (e.g., https://github.com/MineralMate-Program. This will run on Windows machines with the appropriate MATLAB Runtime (Version 9.8 (R2020a) of the MATLAB Runtime).

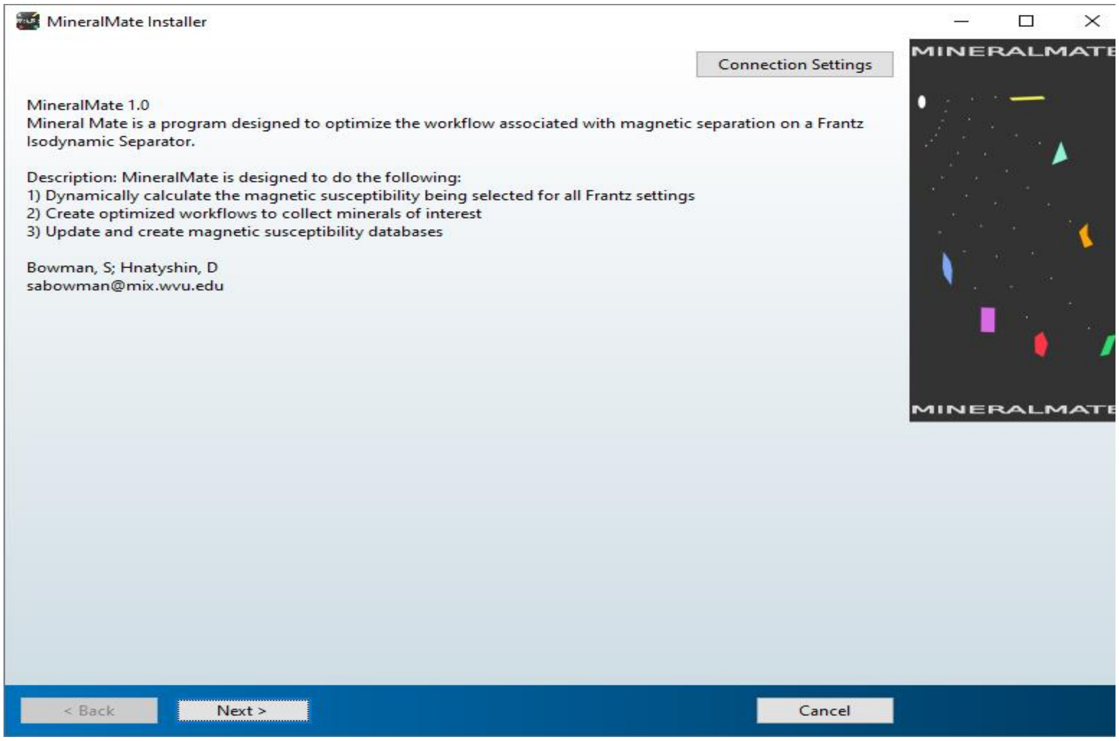

Figure 1: Installer screen for MineralMate, after download of program from one of the repositories.

<u>Operation</u>

Numbered entries 1., 2., 3.,…,etc., herein are stepwise instructions. Bullet points are explanations for the various buttons found in the GUI of the program. Underlined terms correspond to buttons within the program of the same name. Figures containing red boxes are for explanation purposes and do not appear in the program. Additionally, the user may right-click in a field or button in the program for further help if needed.

1) Load Database (**Fig. 2**). If this is the first time using MineralMate, download and save the available Database from one of the repositories (e.g https://github.com/MineralMate-Program). Otherwise, open a Database from your PC.

   Note: It is important that users are advised against modifying the Database itself, but rather conduct modifications *within* MineralMate. These modifications are copied over to the Database, ensuring that its formatting and thus, integration within the program is not adversely affected.

There are four sections (Tabs) to the program. Each section is arranged as a separate tab at the top of the screen. The four Tabs in order from left to right are, Mineral Identification, Workflow Suggestion, Mineral Creation, and Database Modification. Upon opening MineralMate, the default Tab is Mineral Identification.

Mineral Identification-- *3.1 in Bowman and Hnatyshin (2022)*

This Tab is used to determine how the minerals in a sample suite will behave with respect to current or side slope. The output of the Mineral Identification Tab provides the user with an easily identifiable colour-coded reference for each of the minerals in their sample.

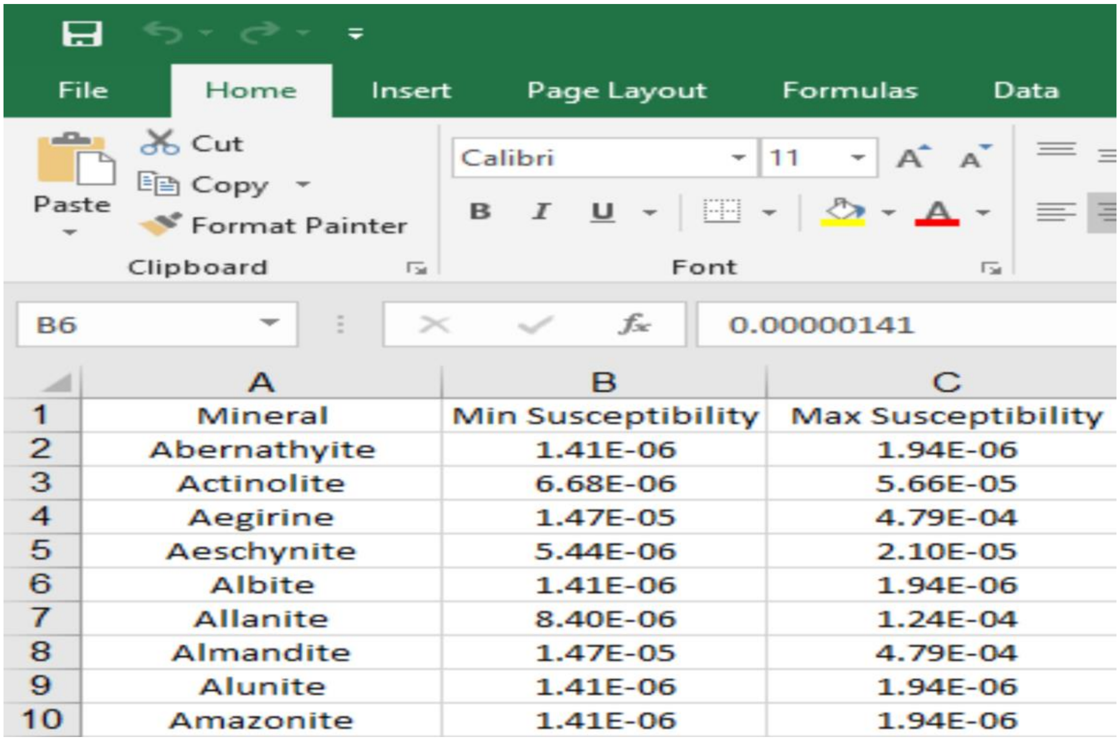

	A	B	C
1	Mineral	Min Susceptibility	Max Susceptibility
2	Abernathyite	1.41E-06	1.94E-06
3	Actinolite	6.68E-06	5.66E-05
4	Aegirine	1.47E-05	4.79E-04
5	Aeschynite	5.44E-06	2.10E-05
6	Albite	1.41E-06	1.94E-06
7	Allanite	8.40E-06	1.24E-04
8	Almandite	1.47E-05	4.79E-04
9	Alunite	1.41E-06	1.94E-06
10	Amazonite	1.41E-06	1.94E-06

Figure 2: Example Database. Note the formatting of columns A, B, and C. MineralMate imports the information from these columns, so it is important that (for example), "Mineral" stays in column A.

Note: Recall that K_m is magnetic susceptibility (degree to which a mineral is attracted to an external magnetic field). The side slope of the magnetic separator is in degrees, and the current in amps, is used to increase or decrease the strength of the electromagnetic field.

- Slope: If Current is ticked, the resulting graphic (**Fig. 3**) will indicate which Minerals are Magnetic (red), Potentially Magnetic (yellow), and Non-Magnetic (blue) as a function of Side Angle. All Minerals are either red, yellow, or blue at a fixed Current, dependent upon the Side Angle. The graphic displays the behaviour that the mineral will exhibit, either entering the magnetic or non-magnetic chute of the magnetic separator. If the mineral is "Potentially Magnetic", then the mineral is likely to enter both chutes of the magnetic separator at the same time.

  Example: If your electromagnet overheats beyond 1.5 A, and you want a mineral (blue) to enter the magnetic chute, you can simply lower the Side Angle such that the mineral is now magnetic (red) at currents below 1.5 A.

- Current: If Slope is ticked, the graphic now displays how the different Minerals in the sample behave as a function of Current. That is, all Minerals are either red, yellow, or blue at a fixed Slope, dependent on the Current. The effect of altering the Current is apparent and can be tailored as desired.

  Example: If the side slope needs to be fixed (unable to rotate wheel, inaccessible, etc) at 10 degrees, then a Non-Magnetic Mineral (blue) may become Magnetic (red) at a Current of 1.4 A.

- Susceptibility: If Susceptibility is ticked, the Min K_m and Max K_m values (using EQ.2 of Bowman and Hnatyshin (2022)) are provided in the box at the top of the screen for the Minerals in the sample. The graphic at the bottom of the screen displays the qualitative magnetic susceptibility (Magnetic, Potentially Magnetic, or Non-Magnetic).

  Note: Recall that (from Bowman and Hnatyshin, 2022),

$$K_m = sin\,(\alpha)\ * 10^{-6}(\frac{I}{4.7884})^{\frac{-1}{0.5142}}, \qquad EQ.2$$

where I is current (A), and side slope is α.

- Add Phase: See the Add/Delete Phase button in the **Database Modification** Tab
- Export: The Export button will save the Mineral-Min K_m-Max K_m-Select table as a Microsoft Excel file. The Select[ed] Minerals will appear as TRUE in column D of the exported file as a reference.

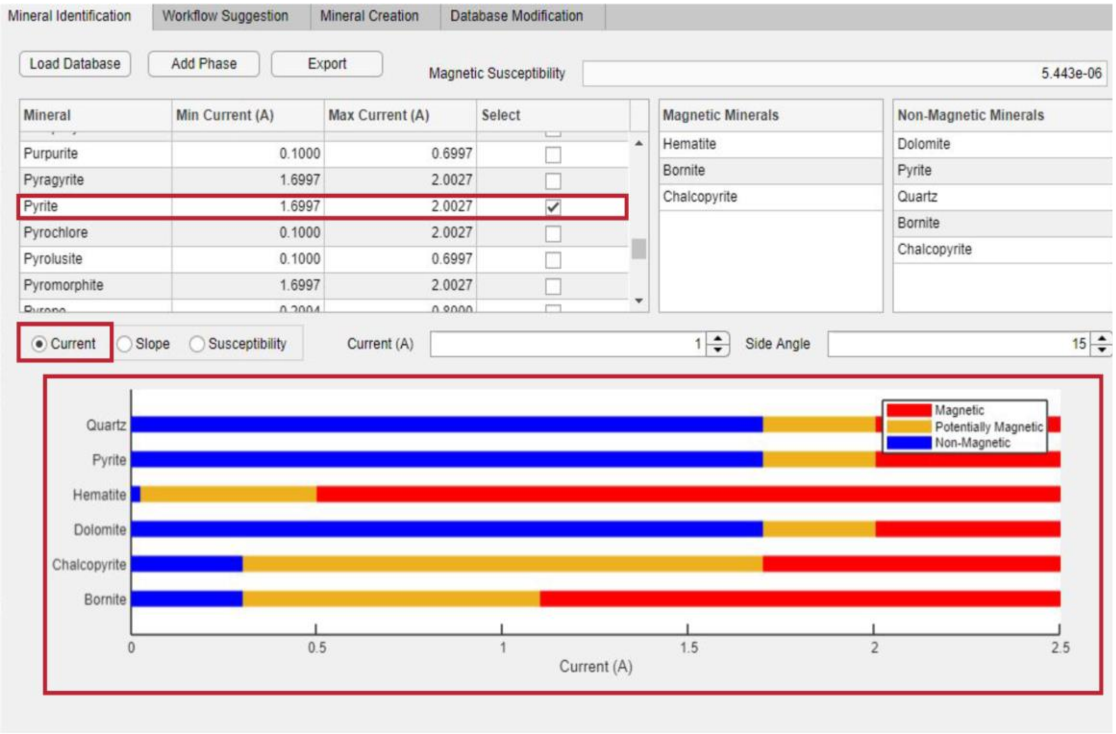

Figure 3: Tab 1 of MineralMate. Selected minerals (e.g., Pyrite), will appear in the table at the top of the screen. Corresponding magnetic behaviour of the Select[ed] minerals will appear in the graphic at the bottom of the screen (Quartz, Pyrite, Hematite, Dolomite, Chalcopyrite, and Bornite). Quartz, Pyrite, and Dolomite have the lowest K_m, and will enter the non-magnetic chute of the magnetic separator at all Currents up to ~1.6 A at 15°, while the other minerals will display some degree of Magnetic behaviour (red or yellow) at Currents less than 1.6 A at 15°.

Workflow Suggestion---*3.2 in Bowman and Hnatyshin (2022)*

This Tab provides the user a stepwise procedure to follow to separate the various mineral phases from their sample. There are two modes from which to choose. The "Workflow Suggestion" provided by MineralMate gives the user a procedure to follow during magnetic separation. By starting with a raw suite of unseparated minerals, MineralMate will provide a suggested sequence that the user can follow to successfully isolate the different mineral phases in their sample.

- Normal mode: The separation begins at the **highest** current (e.g. Step 1 begins at 1.5186 A and a slope of 12° (**Fig. 4)** with subsequent steps occurring at lower currents. Process refers to those Minerals that are to be re-fed through the separator again, while Collect refers to those Minerals that are to be isolated as their separation is complete.

  Tip: Additionally, when first starting mineral separation, the maximum current achievable is likely greater, because the temperature of the electromagnet is lower. It is expected over time that the maximum current achievable will be reduced as the electromagnetic heats up during separation. Therefore, this approach likely grants greater flexibility, especially for relatively non-magnetic minerals. If the user is

concerned about their electromagnet overheating, it may be useful to run their separation in Normal Mode.

- <u>Reverse mode:</u> In this mode the separation begins at the **lowest** current. The current is increased throughout subsequent steps whereby more and more mineral grains are magnetized. In Reverse mode the user collects the "magnetic" separates from the magnetic chute of the separator, while placing "non-magnetic" separates back in the feed of the separator to Process again.
- <u>Load Database:</u> This will load a database of the form shown in **Fig. 4**.

  Note: Alternatively, the Database may be loaded from the **Mineral Identification** tab.

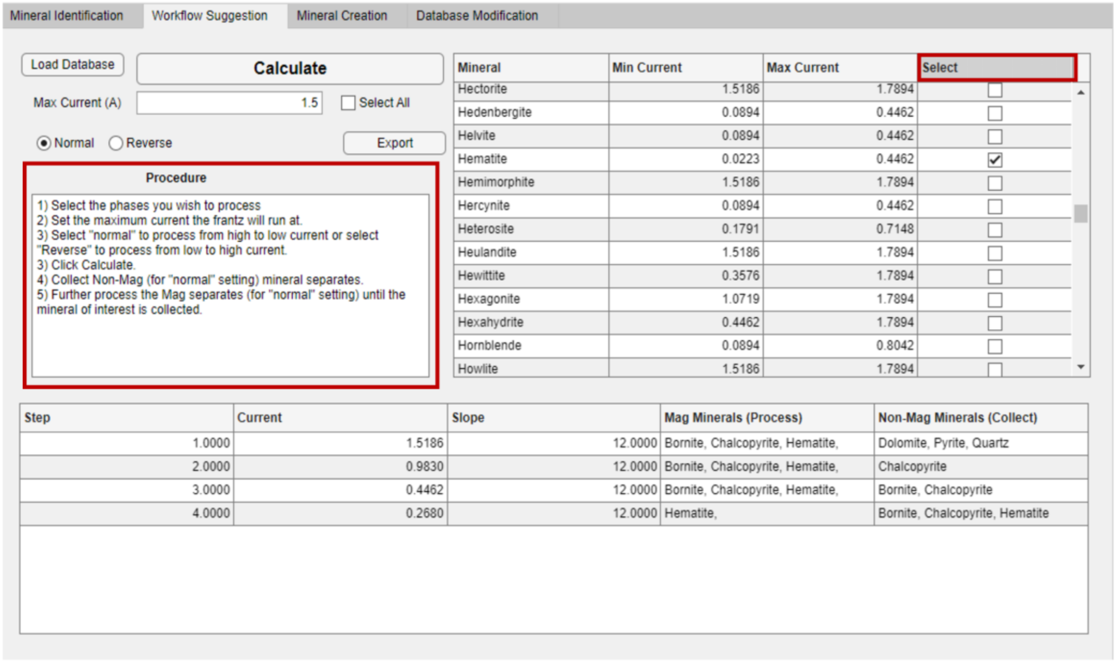

Figure 4: Tab 1 of MineralMate. The Select[ed] minerals (Bornite, Chalcopyrite, Dolomite, Hematite, Pyrite and Quartz), will appear in the menu table at the top of the screen. The pointwise protocol of the Select[ed] minerals in the Procedure box. Step[s] and operating parameters (Current and Slope) along with the mineral behaviour for each Step is provided in the lower window.

Mineral Creation-------------------------------------*3.3 and 3.4 in Bowman and Hnatyshin (2022)*

While the Database contains over 350 minerals, there are numerous mineral species that are not included. If the user encounters one of these minerals, they may utilize this Tab of MineralMate to identify the K_m values of this "new" mineral species. In turn, the user may then upload their findings to the global community. Bowman and Hnatyshin (2022) obtained tested this feature empirically using the mineral forsterite. By constructing a cumulative distribution function (CDF) for forsterite, the K_m values may then be calculated. To construct a CDF for any mineral species, the user would gradually increase the current at a constant side slope (no crystals enter the magnetic

chute) until all of the crystals enter the magnetic chute. The CDF should ideally be monotonic, as current ∝ magnetized crystals.

The Mineral Creation Tab plots the CDF for a mineral with corresponding magnetic weight % values at a given current. These (x,y) pairs form the CDF and may be found for any mineral or crystal (including composite crystals). As mentioned above, the weight % increases to a maximum (100 %) in proportion to the current. This Tab may be used to constrain the K_m values of a mineral that is not currently in the Database, by graphically displaying the exhibited behaviour with respect to various parameters (e.g., Current). It may additionally serve to increase user efficiency by indicating operating conditions that are not favourable for mineral extraction (not normally distributed about some median or mean).

1) Load Mineral: This button requires that the file being loaded has the columns (starting from left to right), Current, Slope, Mag (%), Non-Mag (%), K_m

- Add Datapoint: This button is used to add additional datapoints. The user must provide a current, slope, Mag (%), and Non-Mag (%) value. MineralMate will update the CDF and calculate the corresponding K_m value for the inputted settings. If applicable it will update the estimated minimum and maximum K_m values for this mineral.
- While Current (A) and Mag (%) are used as an example here, other comparisons may be made. Non-Mag (%), K_m, and Slope may be compared to make useful interpretations about a mineral's behaviour.

  Example: K_m (x-axis) vs Non-Mag (%) (y-axis). Both Mag % and Non-Mag (%) may be calculated by using the general form, X (%) = (a (mass)/(a (mass) + b (mass)))*100%. Where a and X may be either Mag or Non-Mag masses, with b being the other. The K_m may then be calculated using EQ.2, or taken from the Database. This approach may be helpful as it graphically displays the range of K_m for a mineral and how the Non-Mag (%) is distributed. Using a Max Mag Cut-off value of 90 % along with a Min Mag Cut-off value of 5 % for the mineral forsterite, the mineral's behaviour can be observed with respect to Current (**Fig. 5**), along with the Min K_m and Max K_m values of 5.99e-5 and 1.18e-4 respectively. Similarly, in **Fig. 6**, the same forsterite sample exhibits a median K_m of ~8e-5.
- Remove Datapoint: Conversely, to remove a coordinate pair (poor fit, impure sample), highlight the row containing the undesired x,y coordinate pair and then click Remove Datapoint (**Fig. 5**).

  Note: Alternatively, the user may untick the x,y data for a Datapoint using the Select button in the table.

  Note: If the information in the table needs to be corrected or changed, simply click on a cell and enter the new value. This change will now be used and reflected graphically in "Mineral Creation".
- Max Mag (%) Cut-off: The CDF plot **(Fig. 5**) may show a skewed distribution. If this is true, the Cut-off edit boxes may be altered to better reflect a more normal distribution about the median 50 wt % (mean = median for normal distribution).

  Tip: Skewness or long tails in the CDF plot could indicate impurity of the sample (either composite grains, or phases of differing mineralogy).
- Export Mineral: Send and save the parameters (Max Mag (%) Cut-off, Min Mag (%) Cut-off, along with the calculated minimum K_m and maximum K_m values) to a Microsoft Excel tab

named "K_m Values", while the ticked Datapoints (Current, Slope, Mag %, Non-Mag (%), K_m, and Select = TRUE) are in a tab named "Datapoints".

Note: To complete the cycle from Mineral Creation to Database addition, the user may simply refer to the .xlsx file containing the information from their new mineral. For example, the user would navigate to the "K_m Values" tab and then copy the Minimum Magnetic Susceptibility (K_m) and Maximum Magnetic Susceptibility (K_m) values from columns A and B respectively *back into* MineralMate's Database (this information would go into the Input Type field of the *Add/Delete Phase* Tab of the **Database Modification** Tab)-see **Fig. 7** (ensure that K_m is ticked).

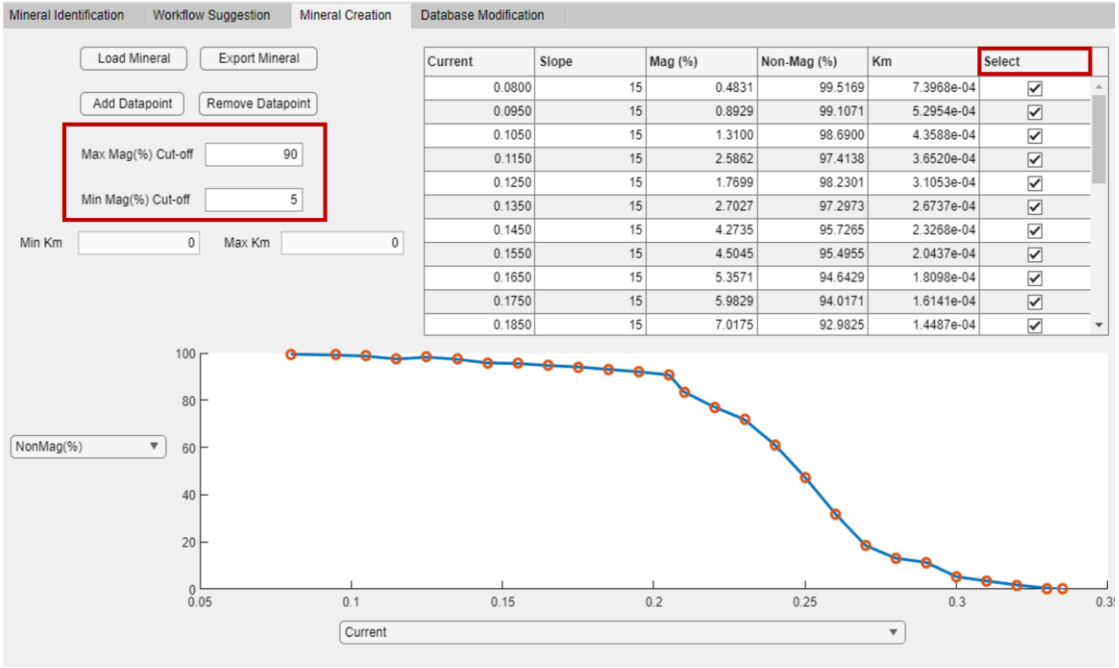

Figure 5: CDF of the mineral forsterite. Forsterite in this sample is distributed about a median (50 wt % NonMag) of 0.25 A.

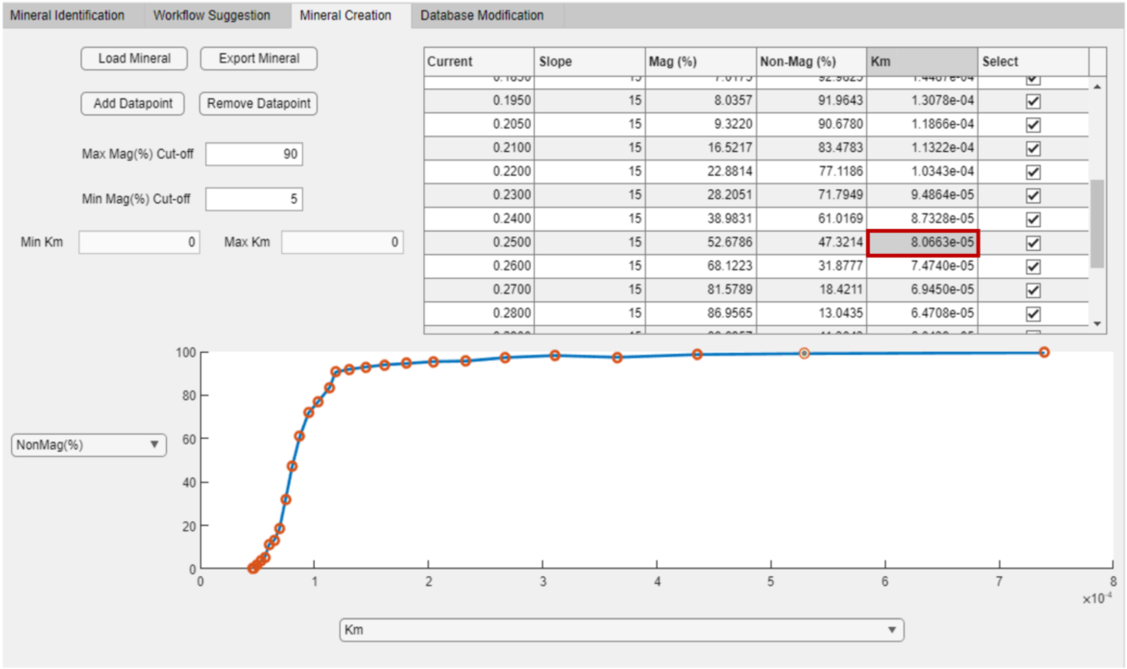

Figure 6: CDF of forsterite comparing NonMag(%) to K_m. The median K_m value of this sample is ~8e-5.

Database Modification---------------------------------*3.3 and 3.4 in Bowman and Hnatyshin (2022)*

This Tab contains two headings (*Add/Delete Phase*, and *Add Composite Phase*) which are outlined independently. It is a good reminder to not alter the Database directly from the Excel file, but to instead make adjustments within MineralMate, and then to Export the new Database. Users may then upload their new Database to the MineralMate Github account (https://github.com/MineralMate-Program) for global sharing.

Add/Delete Phase

1) Phase Name: Enter Phase Name (NewPhase1 in **Fig. 7**) and select either the K_m or Frantz Settings buttons.
2) Enter the values for the Current, Slope if using Frantz Settings, or the Min K_m and Max K_m if using the K_m setting.
3) Update Tables: Your new Phase Name will be added to the Database (NewPhase1) and will propagate throughout the other Tabs within MineralMate. The Min K_m and Max K_m values are calculated using EQ.2 of Bowman and Hnatyshin (2022).

- Delete Selected: If you encounter an error or wish to erase a Phase and start again, simply find the mineral in the table and tick the “Delete?” box, followed by “Delete Selected”.
- Export: Saves and sends the Min K_m and Max K_m of NewPhase1 (**Fig. 7**) to the Database for future separations that include this mineral.

Note: This button is how users update the Database and the resulting .xlsx file will be in the same format as the imported Database. Users are encouraged to upload their Database additions/contributions to the Github MineralMate repository.

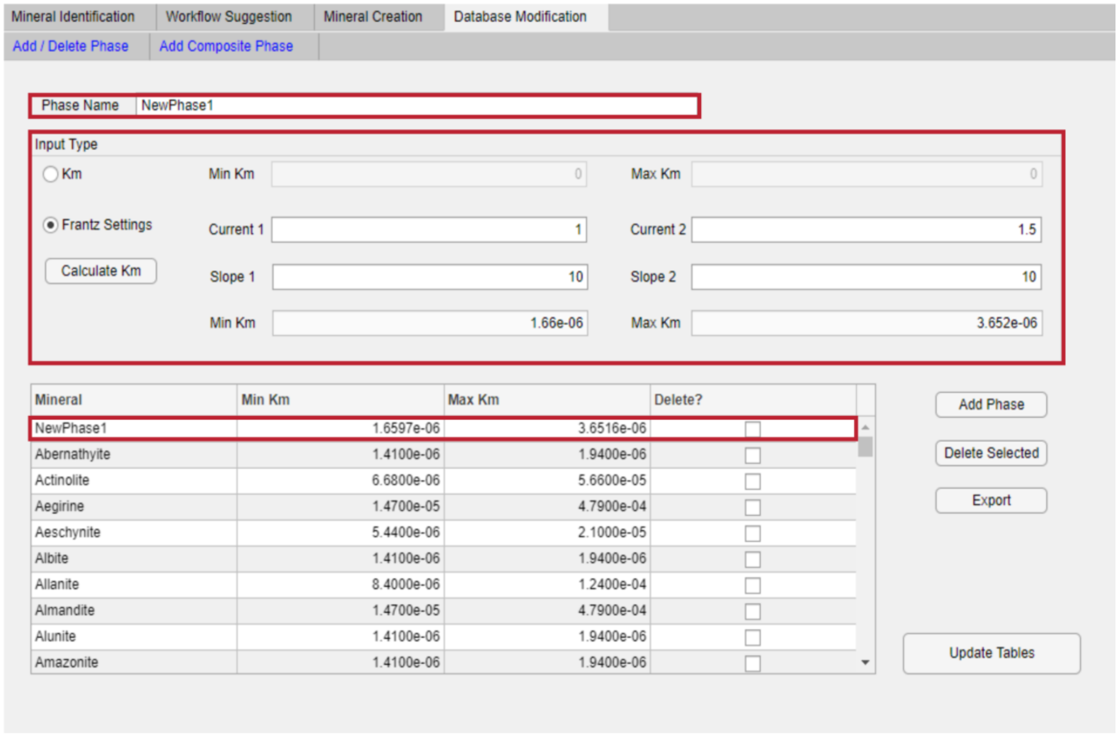

Figure 7: Add/Delete Phase subsection. The new Phase Name is entered at the top left of the screen along with the operating parameters in the Input Type field. The resulting Min K_m and Max K_m values for this NewPhase1 will appear in the table at the bottom of the screen. This information can then be Export[ed] to the Database for future use.

Add Composite Phase

1) <u>Add Composite Phase:</u> Enter Phase Name (NewPhase2; **Fig. 8**) for the composite crystal (e.g. 'user crystal 1)

    Note: This is the composite grain name, not the name of any particular phase *within* the composite grain.

2) Add the various Mineral phases that *are* within the composite crystal using the <u>Select Phase</u> dropdown menu. This menu is a collection of all Minerals in the loaded Database.
3) Enter the Weight (%) of each mineral phase in the table at the top.

    Note: Ensure that the sum of the Weight (%) of all Minerals = 100 % (see **Fig. 8**). The Weight (%) of this composite crystal are approximated from **Fig. 3** in Bowman and Hnatyshin (2022). In this example, if we assume that there are many composite crystals in our sample containing ~5 wt % galena, ~45 wt % pyrite, and ~45 wt % sphalerite, the resulting K_m of the crystal would range from 1.41e-6 to 2.57e-6.

More pyrite-rich composite crystals will be less susceptible (smaller K_m value) than sphalerite-rich composite crystals (greater K_m value).

4) Click <u>Combine and Create</u>, followed by <u>Update Tables</u>. This will now add the composite crystal, NewPhase2, to the table at the bottom of the screen. Additionally, this composite crystal will now be added to the Database. In addition, its accompanying Min K_m and Max K_m values will also be added throughout the other three Tabs in MineralMate.

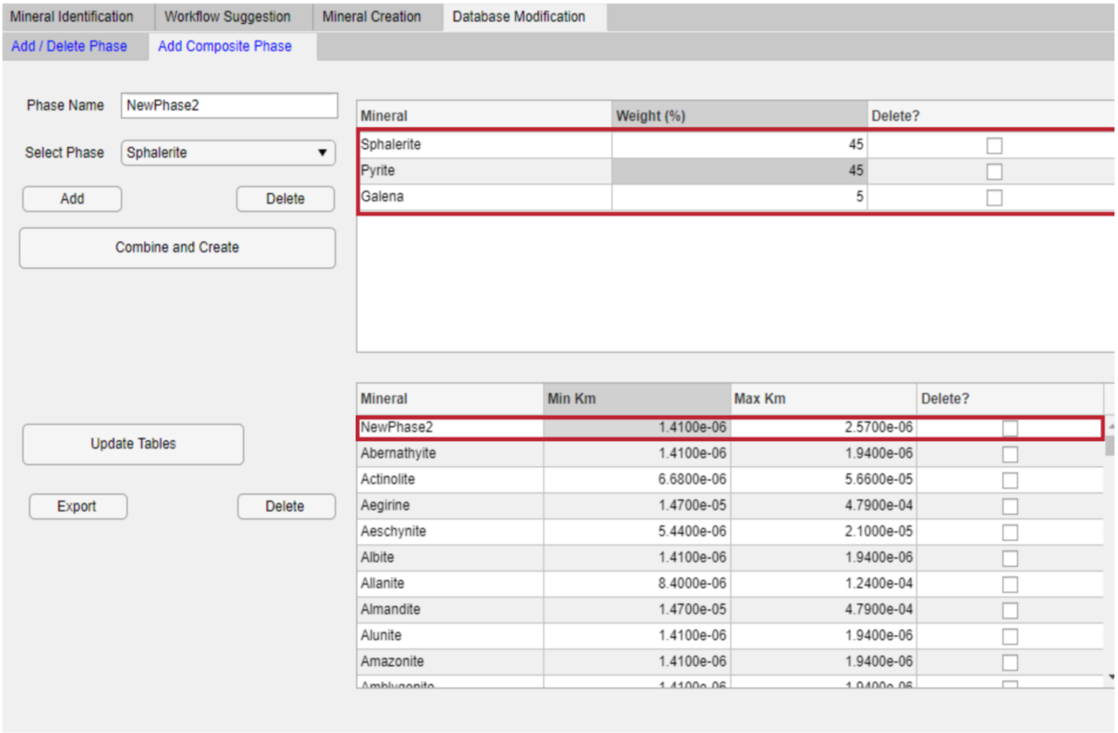

Figure 8: Add Composite Phase subsection. NewPhase2 is a composite crystal composed of three Minerals (5 wt % Galena, 45 wt % Pyrite, and 45 wt % Sphalerite).
