# Supplementary material for: MineralMate: A standalone MATLAB-based aide for the magnetic separation of minerals
Source: Heliyon. 2022 Aug 27;8(9):e10411. doi: 10.1016/j.heliyon.2022.e10411 (PMC9459427; doi:10.1016/j.heliyon.2022.e10411)
Supplement: MineralMate Appendix.docx [file mmc6.docx]

**Appendix**

1.1 β Values

Rearranging EQ.1 for β gives

$$\beta\left( I \right)= \frac{\left( \frac{X*I^{2}}{{10}^{-6}} \right)}{sin \left( \alpha\right)} AEQ.1$$

The variation of β can be determined for a constant slope as a function of I. Taking Χ (or K_m_) obtained from EQ.2 for and substituting into the above equation for β allows for this coefficient to be determined. Data are provided in Appendix Table 1. The average β value of 20.8 ± 0.9 is extremely close to the 20.6 ± 0.7 β value of Nesset and Finch (1980). Additional discussion regarding the sensitivity of β to I is provided below in Appendix Section 1.3.

1.2 Derivation of EQ.2

In Figure 3 of McAndrew (1957), a cross plot in (sin(α)/Χ)*10^-6^ versus I (A) space is made for ferrous ammonium sulfate, nickel sulfate, cupric sulfate, and zinc sulfate (Zeehan sphalerite). A power law relationship was applied to provide a line of best fit for the (x,y) coordinate pairs of the data from McAndrew (1957), where x values are represented by sin(α)/Χ*10^-6^, and y values are represented by the current (I). The fitted power law is given as

$$I=4.7784*\left( \frac{sin \left( \alpha\right)}{X} \right)^{0.5142}*{10}^{-6} AEQ.2$$

which can be rearranged as:

$$\left( \frac{I}{4.7784} \right)^{\frac{1}{0.5142}}=\left( \frac{sin \left( \alpha\right)}{X} \right)*{10}^{-6}.$$

Taking the inverse of both sides gives:

$$\frac{1}{\left( \left( \frac{I}{4.7784} \right)^{\frac{1}{0.5142}} \right)}=\left( \frac{X}{sin \left( \alpha\right)} \right)*{10}^{6}.$$

Solving for Χ = K_m_ gives

$${X=K}_{m}=\left( \frac{1}{\frac{\left( \left( \frac{I}{4.7784} \right)^{\frac{1}{0.5142}} \right)}{{10}^{6}}} \right)*sin \left( \alpha\right). AEQ.3$$

Or, as shown in EQ.2

$${X=K}_{m}=sin \left( \alpha\right) *{10}^{-6}({\frac{I}{4.7884})}^{\frac{-1}{0.5142}}. AEQ.4$$

1.3 Comparison Between EQ.1 and EQ.2

The percent difference between the K_m_ obtained from EQ.1 and EQ.2 were performed for all 379 minerals in the Database. Using β of 20, the difference between these equations converges at I of 0.4 A, and are provided in Appendix Figure 1A-B. Additionally, these percent differences are determined using β of 20.8 and are provided in Appendix Figure 1C-D. β of 20.8 in EQ.1 produces a more similar K_m_ to that found using EQ.2 (lower % difference) for less magnetic minerals. However, β of 20 in EQ.1 produces more similar K_m_ to that found using EQ.2 for more magnetic minerals. Taken together with the results in Appendix Table 1, it is worth noting that β progressively increases with current and that this fitting coefficient is not static. EQ.2, and MineralMate at-large, eliminates the need for the fitting coefficient β, utilizing a low value of β for low currents and a high value of β for high currents may provide better model the K_m_ of the minerals in the sample. Finally, there is overall good agreement between K_m_ obtained using EQ.1 and EQ.2, particularly in the intermediate current range (0.2 A to 1 A for β of 20, and 0.5 A to 1.5 A for β of 20.8).


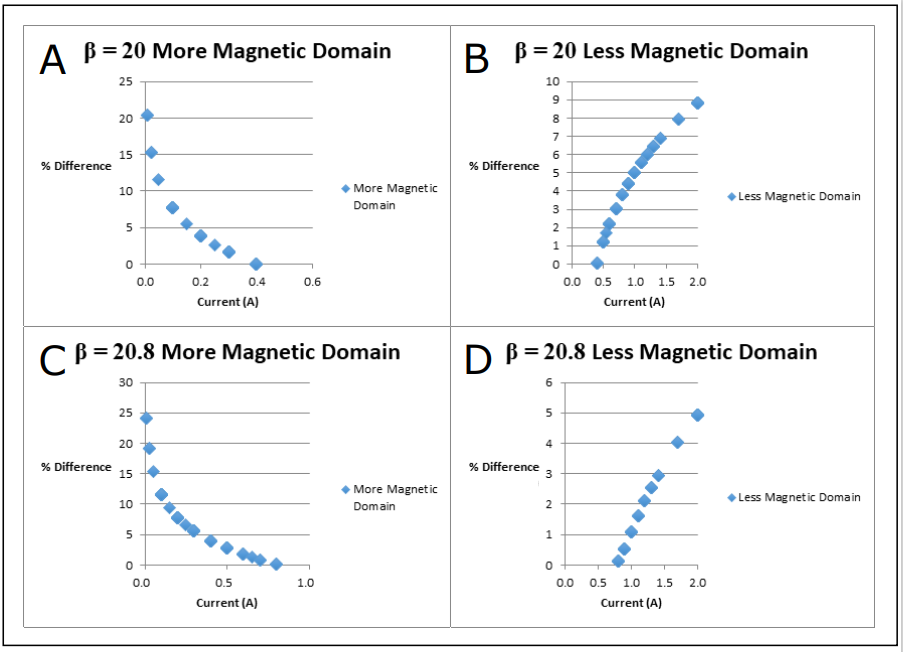


Figure 1: Comparison of the effects of different β on K_m_ for the mineral forsterite. A-B) Percent difference between K_m_ obtained from EQ.1 using β of 20 and K_m_ obtained from EQ.2. C-D) Percent difference between K_m_ obtained from EQ.1 using β of 20.8 and K_m_ obtained from EQ.2.


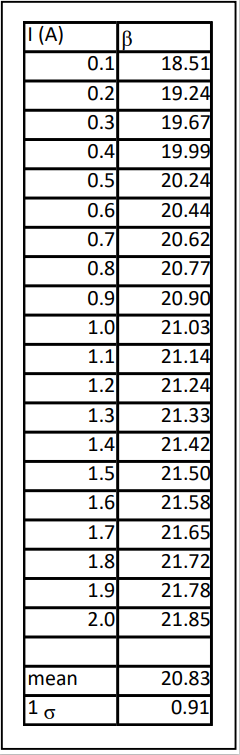


Table 1: β(I) calculated using AEQ.1 and K_m_ from EQ.2 at varying currents.
